# Ecofriendly strategy based on the combination of inoculation with plant growth-promoting bacteria and reduced nitrate doses boosts the valorization of purple cauliflowers

**DOI:** 10.3389/fpls.2025.1571301

**Published:** 2025-05-29

**Authors:** Jacinta Collado-González, María Carmen Piñero, Ginés Otálora, Yamara González, Josefa López-Marín, Francisco M. del Amor

**Affiliations:** Department of Crop Production and Agri-Technology, Murcia Institute of Agri-Food Research and Development (IMIDA), Murcia, Spain

**Keywords:** antioxidant compounds, nitrogen dose, ecofriendly strategy, valorization of purple cauliflower by-products, plant growth promoting bacteria

## Abstract

**Introduction:**

The reduced N doses in plant fertilization, combined with the influence of plant growth-promoting bacteria (PGPB), to obtain ecofriendly crops and their derived products can be a significant challenge. Purple cauliflower is an exotic variety that can generate higher profits for farmers and greater interest among consumers seeking novel and healthy foods. Purple cauliflower florets are edible, while the leaves are discarded because they are by-products.

**Methods:**

The aim of this research was to investigate the combined effect of PGPB (*Azotobacter salinestris* strain CECT9690) and three different N doses supplied in the nutrient solution (30%, 60%, and 100%) on plant growth and the quality of florets and by-products. Plant height, total shoot and floret weight, and % dry weight in florets and leaves were improved by A. *salinestris* inoculation under limited doses of N.

**Results and discussion:**

The sugar content in the leaves of plants grown with a limited N dose (30%) and inoculated with PGPB increased by 51% compared to the control. PGPB inoculation increased proteins in the leaves (by 30% with 100% N supply, 33% with 60% N supply, and 16% with 30% N supply). Additionally, PGPB inoculation enhanced potassium (26%) and iron (34%) in leaves of plants under limited N supply. These findings indicate that the combined use of reduced N supply and PGPB inoculation may be considered an ecofriendly strategy to enhance the growth and quality of purple cauliflower, while boosting the valorization of its by-products.

## 1 Introduction

Cauliflower (*Brassica oleracea* L. var. botrytis) is a vegetable belonging to the Brassicaceae family, which plays an important role in human nutrition and potentially in health (Shinali et al., 2024). The production of cauliflower, along with broccoli, exceeded 26 million tons worldwide in 2022. The main producers of these crops in 2022 were Asia (81%), Spain and Italy (8.3%), and the Americas, including the United States and Mexico (8.2%) (FAO (Food and Agriculture Organization of the United Nations), 2025). According to FAO (2022), the agricultural area dedicated to these crops in the Region of Murcia (SE Spain) amounts to 13,340 ha with a production of 18,830 kg/ha, representing more than 45% of Spanish production of this vegetable. Although white cauliflower is the most commonly consumed variety, those of other colors are receiving increasing attention. In fact, the colorful varieties are enriched with different nutrients that give them their colors. Furthermore, these exotic varieties are promising both for farmers, who seek higher profits, and for consumers, who desire healthy foods (Singh et al., 2020).

Brassicas, in general, owe their popularity to their high contents of vitamin C, proteins, minerals, glucosinolates, and their derivatives, isothiocyanates and sulforaphane (Kaiser et al., 2021). Brassicas are also very rich in phenolic compounds (Shinali et al., 2024). Purple cauliflower owes its purple color to a high content of anthocyanins, which is important for humans as these are among the most effective antioxidants, reducing enzymatic activity and exhibiting significant anti-mutagenic and anti-carcinogenic effects (Luo et al., 2023). This provides a preventive effect against atherosclerosis, venous insufficiency, cardiovascular diseases, certain types of cancer, and other chronic diseases (Singh et al., 2020).

It is important to note that not all parts of the cauliflower plant are edible, with a significant portion considered disposable waste (Collado-González et al., 2021a). The accumulation of bioactive compounds in different plant tissues could be of interest to the cosmetics industry, as it allows for the utilization and profitability of these by-products through their valorization, promoting their use for nutraceutical purposes and enhancing the sustainability of various crops (Mora-Garrido et al., 2022). Additionally, the valorization of such by-products would be associated with greater sustainability, providing them with added value in the current market. This is crucial considering that today’s consumers not only seek healthy products but also demand that they be sustainable products (Gallo et al., 2023).

Numerous studies have reported that an adequate supply of nitrogen (N) to plants stimulates their growth, development, and the expression of genes involved in flavonoid synthesis, promoting their accumulation and enhancing antioxidant activity in most plants (Stefaniak et al., 2020). However, an excessive supply of N to plants can lead to the opposite effect, resulting in plants with poor concentrations of bioactive compounds. This undesirable effect is compounded by an increase in harmful nitrate, which is damaging to the ecosystem and harmful to humans (Collado-González et al., 2024). Although an excessive supply of N in the form of nitrate can be detrimental to plants, a severe deficiency of N can also produce undesirable results, such as smaller plants with poor nutritional quality (Munene et al., 2017; Collado-González et al., 2024). For this reason, in recent years, the study and use of plant growth-promoting bacteria (PGPB), which are N-fixing, have gained importance. The use of PGPB improves crop yields under optimal or suboptimal growth conditions, avoiding excessive use of N-based fertilizers and resulting in energy savings, increased commercial profits, and reduced environmental impact (Consentino et al., 2022). Among the several N_2_-fixing bacteria, *Azotobacter* species are well known because they are capable of combining nutritional aspects related to plant growth and development, improving resilience that determines resistance to harsh environmental conditions or abiotic stress (Aasfar et al., 2021).

Shrestha et al. (2022) stated that cauliflowers inoculated with *Azotobacter* and fertilized with a full dose of nitrogen grew larger and showed better yield. However, Kumar et al. (2024a) reported that the integrated use of chemical fertilizers (75% NPK), *Azotobacter* bacteria, and plant growth hormones had a significant effect on the growth parameters and yield of broccoli. Consentino et al. (2022) found that the inoculation of PGPB enhanced plant growth, productivity, and nutritional quality of lettuce, mainly under low nitrogen doses (50% compared to the control treatment). Collado-González et al. (2024) indicated that the inoculation of celery plants with *Azotobacter* under a nitrogen-deficient regime (60% and 30%) resulted in plants with improved nutritional quality.

The starting hypothesis of this work is that inoculation with PGPB may alter several growth and nutritional quality parameters of purple cauliflower under reduced N doses. This alteration could increase the tolerance/resilience of the purple cauliflower plant to nutritional stress due to N deficiency and could also have beneficial effects on its florets and leaves. Thus, this strategy could also promote the valorization of its by-products. Therefore, the purpose of this work is to study how inoculation with *Azotobacter salinestris*, a PGPB, affects the biomass, antioxidant activity, total phenolic compounds, and accumulation of macronutrients, micronutrients, sugars, and proteins in purple cauliflower florets and leaves (by-products), under limited N doses. Based on previous studies, the N doses supplied in the nutrient solution will be the control (100% N), a moderate N deficiency (60% N), and a severe nitrogen deficiency (30% N).

## 2 Materials and methods

### 2.1 Experimental design and treatments

The experiment was conducted on cauliflower variety Graffiti F1 (El Jimenado S.A, El Jimenado, Murcia, Spain). Thirty days after sowing, the seedlings were randomly transplanted into 1.2-m-long bags (22 L) filled with coconut fiber (supplied by Pelemix, Alhama de Murcia, Murcia, Spain). Each bag had three plants and three drippers (2 L h^-1^). Consecutive bags were spaced 30 cm apart within rows, with 1 m between rows, in a polycarbonate greenhouse located in the region of Murcia (37°56’27.3” N, 1°08’01.8” W). The experiment lasted 92 days under controlled conditions, with a temperature regime of 28/15°C and a relative humidity of 70%. Three fertilizer treatments were used: control treatment (100% of N supply), another one that had a mild deficiency of N (60% of N supply) and the last one with a severe deficiency in N supply (30% of N supply). A modified Hoagland’s nutrient solution was used for the irrigation of control treatment. That Hoagland’s nutrient solution was composed of Ca(NO_3_)_2_·4H_2_O (362.0 mg L^-1^), KNO_3_ (404.4 mg L^-1^), K_2_SO_4_ (131.1 mg L^-1^), MgSO_4_·7H_2_O (123.2 mg L^-1^), H_3_PO_4_ (0.101 mL), and micronutrients. Each fertilizer treatment was applied to 48 plants (8 blocks with 6 plants each one). After transplanting, half of the plants in each fertilizer treatment (24 plants per treatment) were inoculated with the *A. salinestris* strain CECT9690 (1 x 10^7^ CFU/g), obtained from Ceres Biotics Tech, S.L. (Madrid, Spain). Following the manufacturer’s recommendations, 10 mL of inoculant (250 μg mL^-1^) were added to each plant and mixed uniformly with the substrate.

On the final day of the experiment, six plants per treatment randomly chosen were harvested and divided into two parts: edible (florets) and by-product (leaves). Samples from these parts were frozen at -80°C for antioxidant activity analysis, and other samples were freeze-dried for analyzing sugars, total phenolic compounds, total protein content, and macro- and micronutrients.

### 2.2 Plant biomass measurements

On the final day of this assay, the height of the purple cauliflower plants was measured. Afterwards, plants were collected, their fresh total weight was measured and the plants were separated into edible (floret) and non-edible parts (Serret et al., 2018). The edible part was also weighed. Then, the floret and leaves were lyophilized for 4 days and their dry weight was estimated by weighing these samples again.

### 2.3 Chemical and reagents

Authentic standards for sugars (glucose, sucrose, fructose), gallic acid, and 6-hydroxy-2,5,7,8-tetramethylchroman-2-carboxylic acid (Trolox) were purchased from Sigma-Aldrich (Steinheim, Germany). Folin-Ciocâlteu reagent, sodium carbonate, methanol, nitric acid, hydrogen peroxide, and hydrochloric acid were acquired from Panreac Química (Barcelona, Spain), and acetone from Scharlau (Barcelona, Spain). SPE cartridges (C18 Sep-Pak cartridges) were purchased from Waters Associates (Milford, Mass.), and the ultrapure water used was obtained from a Millipore water purification system.

### 2.4 Total phenolic compounds and antioxidant activity (ABTS^+•^) analysis

The Folin-Ciocâlteu colorimetric method was used for determining the total phenolic compounds (TPC) (Kähkönen et al., 1999). Fresh tissues (0.5 g) were homogenized with acetone (5 mL) (80%, *v/v*), and the homogenates were centrifuged at 10,000 × g at 4°C for 10 min. The supernatant (100 µL) was mixed with a solution of Folin-Ciocâlteu reagent (1 mL) diluted with Milli-Q water (1:10) and 2 mL of Milli-Q water. Then, it was incubated at room temperature (3 min), mixed with sodium carbonate (5 mL) (20%, *w/v*), and shaken vigorously. This mixture was re-incubated in the dark at room temperature for 60 min. The absorbance of the resulting blue-color solution was measured at 765 nm using a UV–visible spectrophotometer (Shimadzu UV-1800 model with the CPS-240 cell holder, Shimadzu Europa GmbH, Duisburg, Germany). The TPC quantification was performed using a calibration curve of gallic acid and results were expressed as mg of gallic acid equivalents (GAE) 100 g^-1^ FW.

The ABTS^+•^ methodology described by Cano-Lamadrid et al. (2017) was followed for analysis of the antioxidant activity. Lyophilized and powdered different tissues of purple cauliflower (0.5 g) were mixed with a solution (10 mL) of MeOH (80:20, *v/v*) + 1% HCl, sonicated at 20°C (15 min), and stored at 4 °C (24 h). Each extract was re-sonicated and centrifuged at 10,000 g at 4°C for 10 minutes. An aliquot of supernatant (10 μL) was mixed with a prepared ABTS+• radical solution (990 µL), shaken, and incubated in darkness (10 min). Then, its absorbance was measured at 734 nm using a UV–visible spectrophotometer (Shimadzu CPS-240 model, Kyoto, Japan). For a satisfactory quantification of antioxidant activity, a Trolox calibration curve was used and the results were reported as µg Trolox equivalents (TE) 100 g^−1^ DW.

### 2.5 Determination of cations

For the determination of cations (Na, Ca, K, Mg, B, Cu, Fe, Mn, P, and Zn) of different tissues of purple cauliflowers the procedure of Piñero et al. (2019) was followed. Briefly, ground freeze-dried samples (0.1 g) were weighed and then digested in 8 mL of nitric acid (concentrated): water (1:1 *v/v*) and 2 mL of hydrogen peroxide, in an ETHOS ONE microwave digestion system (Milestone Inc., Shelton, CT, USA), for 60 min. Then, the digests were diluted to 25 mL with miliQ water and the analysis of the cations content was carried out using a Spectro GENESIS inductively-coupled plasma (ICP-OES) spectrometer (SPECTRO/Ametek Analytical Instruments GmbH, Kleve, Germany). The contents of macronutrients were expressed as g kg^-1^ DW and of micronutrients as mg kg^-1^ DW.

### 2.6 Determination of sugar contents

For extraction of the sugars contained in purple cauliflower, lyophilized tissues (50 mg) were homogenized with an extractant solution of MeOH (80% *v/v*) (1.5 mL). The homogenate was incubated at 4°C for 30 minutes and centrifuged at 3,500 × g at 4°C, for 15 minutes. The supernatants obtained were filtered through a C18 Sep-Pak cartridge (Waters Associates, Milford, Mass.) and combined and re-filtered through a 0.45-μm filter (Millipore, Bedford, MA, USA). The pellet of each sample was re-extracted following the same steps of incubation, centrifugation, and filtering (Balibrea et al., 2003). Both filtrates of the same sample were combined and analyzed (20 μL) using ion chromatography with an 817 Bioscan system (Metrohm, Herisau, Switzerland) equipped with a pulsed amperometric detector (PAD) and a gold electrode, utilizing a METROHM Metrosep Carb 1–150 IC column (4.6 × 250 mm), heated to 32°C. Authentic standards were used for the calibration curve and results were expressed in g·kg^-1^ DW.

### 2.7 Determination of proteins contents

The total proteins content in purple cauliflower was evaluated following a procedure previously described (Kenneth, 1990). For that, freeze-dried samples (after at least 72 h at 65°C) that were analyzed in a combustion nitrogen/protein determinator (LECO FP-528, Leco Corporation, St. Joseph, MI, USA). The crude protein values were obtained as mineral N multiplied by the protein factor (6.25) and the results were expressed as g 100 g^-1^ DW.

### 2.8 Data analysis

The experiment designed was completely randomized with six replications per treatment (n= 6) and with a 3 x 2 factorial scheme, which was composed by three N doses supplied (100%, 60%, and 30%) and two treatments with PGPB (inoculated and non-inoculated plants) as the main factors. The results are presented as the mean ± standard error and all of the data were analyzed with the SPSS software v.25 (IBM, Chicago, IL, USA). The normality of the distribution and homogeneity of variance were tested using the Kolmogorov-Smirnov and Levene tests, respectively. After that, data were also subjected to two-way analysis of variance (ANOVA) and afterwards Tukey’s multiple-range test was utilized to compare the means. Differences were considered statistically significant at p ≤ 0.05.

## 3 Results and discussion

### 3.1 Biomass parameters

The height of the purple cauliflower plants was 52.1 cm, while the fresh weights of the total plant and edible part were 1125.5 g and 337.4 g, respectively (**Table 1**). According to Kumar et al. (2024b), the purple cauliflower variety Graffiti F1 is smaller than the variety Valentena. **Table 1** also shows that the dry matter content in the floret and leaves was 11.4% and 22.4%, respectively. These values are in line with those found for cauliflower, since raw purple cauliflower floret usually contains about 10% dry mass and cauliflower leaves around 20% (Chakraborty, 2016; Kapusta-Duch et al., 2019).

**Table 1:** Effect of the combination of inoculation with plant growth promoting bacteria and three different N concentrations in the nutrient solution on purple cauliflower biomass parameters.

Treatment		Height (cm)	Total shoot weight (g)	Floret weight (g)	% Dry floret weight	% Dry leaves weight
100% N	Without PGPB	50.0 ± 2.1^a^	1125.5 ± 130.9^a^	337.4 ± 65.6^a^	11.4 ± 1.1^a^	22.4 ± 0.6^a^
	With PGPB	52.1 ± 1.92^A^	1270.3 ± 106.1^A*^	531.9 ± 77.1^A*^	13.1 ± 0.8^A*^	24.4 ± 0.9^A*^
60% N	Without PGPB	39.9 ± 3.4^b^	577.6 ± 89.2^b^	194.5 ± 20.7^b^	9.3 ± 0.9^b^	20.2 ± 1.3^b^
	With PGPB	42.9 ± 1.2^B^	625.4 ± 32.51^B*^	237.1 ± 20.6^B*^	11.2 ± 0.9^B*^	22.5 ± 0.6^B*^
30% N	Without PGPB	36.3 ± 1.8^b^	396.6 ± 45.8^c^	153.5 ± 7.9^c^	7.9 ± 1.0^b^	18.9 ± 0.9^c^
	With PGPB	37.8 ± 3.1^C^	432.7 ± 15.0^C*^	176.9 ± 11.4^C*^	10.7 ± 1.3^B*^	21.4 ± 0.3^C*^
Significance						
Nitrogen (N)		***	***	***	***	***
PGPB		**	**	***	***	***
N × PGPB		***	**	***	***	***

The data are presented as the treatment means (n = 5). Different lower case letters indicate significant differences between purple cauliflower plants under different dose of N supplied at p = 0.05 (Tukey’s test). Different upper case letters indicate significant differences between purple cauliflowers grown with or without PGPB Asterisks indicate significant differences between inoculated and non-inoculated plants fertilized with the same N dose. Analysis of variance:

ns, not significant; ** p ≤ 0.005; *** p ≤ 0.001. Abbreviations: No PGPB, plants not inoculated with PGPB.

The cauliflower biomass parameters (i.e.; plant height, total shoot fresh weight, edible part fresh weight, floret and leaves dry matter %) were significantly affected by the N dose supplied in the nutrient solution and the inoculation of plants with PGPB (**Table 1**). The analysis of variance exposed that these factors can present an individual or interactive effect. All growth parameters experienced a drastic reduction as the N supply was reduced. These findings are in accord with experiments on other species, including sorghum, corn, olive, and lettuce (Hong et al., 2022; Jarquín-Rosales et al., 2022), and are expected, since N is a constituent of many biomolecules, including amino acids, amides, proteins, nucleic acids, nucleotides, and coenzymes. Therefore, the plant growth reduction depends upon the extent to which N deficiency is imposed (Bianco et al., 2015). Moreover, Metwaly (2017) stated that to optimize cauliflower plant growth, it was necessary to optimize the doses of P and K along with the dose of N. Thus, a good balance of these nutrients can positively affect plant growth, yield, and nutritional quality.

Our outcomes are supported by those of Singh et al. (2014), who, when using some PGPB, such as *Azospirillum* and *Azotobacter*, found a beneficial effect of the application of these biofertilizers on broccoli plant growth parameters. It has also been shown that PGPB application considerably improves the plant growth traits and dry matter percentage in several crops, including potato and tomato (Consentino et al., 2022). The positive effect of PGPB on plant growth can occur through various pathways, such as N fixation, inorganic phosphate solubilization, and even the ability of PGPB to synthesize hormones crucial for plants (Consentino et al., 2022; Gupta et al., 2022).

### 3.2 Antioxidant capacity (ABTS^+^•) and total phenolic compounds

**Figure 1:**
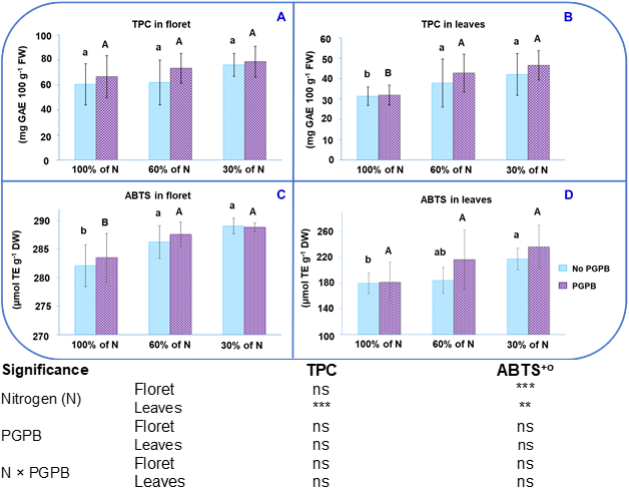
Effect of the combination of inoculation with **(A)** salinestris and three different N concentrations on the total phenolic compounds (TPC) of the floret **(A)** and leaves **(B)** and the antioxidant activity (ABTS) of the floret **(C)** and leaves **(D)**. The data are presented as the treatment means (n = 5). Different lower case letters indicate significant differences between purple cauliflower plants fertilized with different N% supplies, at p = 0.05 (Tukey’s test). Different upper case letters indicate significant differences between purple cauliflower plants fertilized with different N% supplies and inoculated with PGPB. Analysis of variance: ns, not significant; **p ≤ 0.005; ***p ≤ 0.001. No PGPB, plants not inoculated with PGPB.

The values obtained for antioxidant activity (AA) in this study were 282.1 µmol TE g^-1^ DW in floret and 179.8 µmol TE g^-1^ DW in leaves. The TPC values were 60.5 mg GAE 100 g^-1^ FW in florets and 31.3 mg GAE 100 g^-1^ FW in in leaves (**Figures 1A–D**). These TPC values are slightly higher than those obtained in purple cauliflower by Cheng et al. (2021). These authors found that the floret of purple cauliflower had TPC and AA values intermediate among six purple plants (purple cauliflower, purple potato, purple lettuce, purple carrot, purple beans, and purple tomato). Taking into account that the molecular weight of Trolox is 250.29 g mol^-1^, it can be seen that the values shown in **Figures 1A and C** are lower than those found in a commercial purple cauliflower (Cheng et al., 2021). Volden et al. (2009) reported that the Graffiti F1 cultivar has strong antioxidant activity. In this sense, considering that the moisture percentage of the Graffiti F1 cauliflower is 90-92%, our results are similar to those observed by these authors, who indicated that this variety showed an AA close to 26 μmol Trolox equivalents per g of fresh vegetable weight. In another study performed with the same cultivar of purple cauliflower, values lower than ours were recorded (Kapusta-Duch et al., 2019). Some studies have highlighted the impact that the type of crop, cultivar, and plant part analyzed have on the accumulation of bioactive compounds and, consequently, on antioxidant activity (Bhandari and Kwak, 2015). Moreover, the accumulation of these antioxidant compounds in cauliflower is also greatly influenced by the transplanting time, altitude, climatic conditions, and the nitrogen-potassium (N:K) ratio applied to the crop (Toscano et al., 2019; Tallarita et al., 2024).

Contrary to expectations, the TPC content in the floret of purple cauliflower was lower than that found in different varieties of white cauliflower (Bhandari and Kwak, 2015; Collado-González et al., 2021b). Nonetheless, the AA was indeed higher in purple cauliflower than in white cauliflower. This highlights the fact that the antioxidant compounds in purple cauliflower, in addition to the phenolic compounds, include vitamin C, carotenoids, glucosinolates, and tocopherols (Diamante et al., 2021; Tallarita et al., 2024). The TPC values found in purple cauliflower leaves (**Figure 1B**) were similar to those found in the leaves of various white cauliflower varieties (Asia white, Guideline F1, and Moonshine) (Bhandari and Kwak, 2015; Collado-González et al., 2021a; Drabińska et al., 2021). The fact that the leaves show lower AA and TPC contents (**Figures 1B, D**) than the florets supports the theory developed by Tsunoda et al. (2017). This theory suggests that the highest concentrations of bioactive compounds, which are defensive compounds in plants, will be found mainly in the most important and vulnerable organs.

When the different doses of nitrate applied are considered, the antioxidant capacity values obtained were from 282.1 µmol TE g^-1^ DW to 289.0 µmol TE g^-1^ DW in the florets and between 179.8 µM TE g^-1^ DW and 217.6 µmol g^-1^ DW in the leaves (**Figures 1C, D**). The TPC values varied from 60.5 mg GAE 100 g^-1^ FW to 76.0 mg GAE 100 g^-1^ FW in the florets and from 31.3 mg GAE 100 g^-1^ FW to 42.0 mg GAE 100 g^-1^ FW in the leaves. Whilst an increase in TPC was observed in purple cauliflower leaves, an important increase in AA was obtained in both parts of the plant (floret and leaves) as the N supply decreased. **Figures 1A and B** show that the highest TPC in leaves and AA in florets were achieved in plants fertilized with a nitrate dose of 30%. **Figure 1D** illustrates that the highest AA in leaves was achieved in plants with mild or severe N starvation. This increase with low doses of N can be explained by the positive relationship between restricted N fertilization and the biosynthesis/accumulation of phenolic compounds (Stefaniak et al., 2020). Although previous studies report that the maximum accumulation of phenolic compounds in some plants was achieved under severe N deficiency, there are still no conclusive studies on what the optimal N application should be to ensure the highest amount of phenolic compounds without negatively affecting production (Stefanelli et al., 2010).

Regarding the effect of plant inoculation with *A. salinestris* on AA and TPC, it has been found in studies performed on various plant species, such as medicinal and aromatic plants and celery, where a greater accumulation of phenolic compounds compared to reference plants was observed (Chiappero et al., 2021; Collado-González et al., 2024). In this work we have observed a trend toward higher accumulation of TPC and antioxidant activity. To our knowledge, bacterial metabolic activity can play a key role in the stress response, leading to better plant responses to stress. The inoculation with PGPB alleviates plant oxidative stress and enhances the plant tolerant to abiotic stresses through various mechanisms consistent in enzymatic and non-enzymatic responses (Abdel Latef et al., 2020; Sarker and Oba, 2020; Rossi et al., 2021; Neshat et al., 2022). Enzymatic antioxidants, including peroxidase, ascorbate peroxidase, catalase, superoxide, glutathione peroxidase, superoxide dismutase, glutathione-S-transferase, monodehydroascorbate reductase, and dehydroascorbate reductase, can transform superoxide radicals (O 2 ˙−) into hydrogen oxides, which in turn is converted into hydrogen peroxide (H_2_ O_2_) and oxygen (O_2_). Among non-enzymatic antioxidant compounds are chlorophylls, proline, ascorbic acid, cytokinin, gibberellins, indoleacetic acid, ACC deaminase, abscisic acid, trehalose, volatile organic compounds, ethylene, exopolysaccharides, siderophores carotenoids and tocopherol and they also play important roles in scavenging the toxic ROS (Ren et al., 2019; Rossi et al., 2021; Ajijah et al., 2023; Piñero et al., 2023). Despite the observed trend and due to the standard deviation, no statistically significant differences were obtained due to the inoculation or its combination with N restriction. This is in line with what was found in an experiment carried out on lettuce by Ottaiano et al. (2021), who stated that the application of PGPBs at different N levels did not affect either AA or TPC. It is important to taking into account that the effect of PGPB on plants can vary between different laboratory, greenhouse, and field trials. This is due to the effectiveness of PGPR can be affected by the applied agronomic (Ahemad and Kibret, 2014). Numerous studies have reported that PGPR promote plant growth and development through two pathways: direct and indirect. Through the direct pathway, they facilitate the acquisition of resources (nitrogen, phosphorus, and essential minerals) or modulate plant hormone levels. Whilst through the indirect pathway, they act as control agents by reducing the inhibitory effects of various biotic or abiotic stresses on plant growth and development (Ahemad and Kibret, 2014; Abdel Latef et al., 2020; Lephatsi et al., 2021; Neshat et al., 2022). A mechanistic explanation of the results obtained in the current work on the accumulation of antioxidant compounds could be due to: i) both non-enzymatic and enzymatic antioxidant compounds provide a complex and multifaceted protective mechanism to control redox homeostasis to avoid or alleviate oxidation-induced damage in plant cells and support plant development. This complex mechanism involves several factors, such as genotype effects, environmental conditions, the plant’s water and nutrient status, and the type of associated bacteria (Del Amor et al., 2009; Soares et al., 2019; Rossi et al., 2021). ii) It is possible that this strain did not produce as strong an overexpression of antioxidant compounds as expected, possibly because the inoculation with this PGPB strain did not cause a significant alteration in the cell’s metabolism, resulting in a not-so-high antioxidant content, which contrasts with the antioxidant enzymatic system against lipid peroxidation (Di Mola et al., 2019). iii) A limited assimilation of macro- and micronutrients in plants inoculated with PGPB, which could hinder the biosynthesis of amino acids like phenylalanine and tyrosine. These amino acids are specific precursors in the biosynthesis of simple phenolic compounds and flavonoids in the plant cell (Feduraev et al., 2020).

### 3.3 Macro and micronutrients

Ten macro and micronutrients have been studied (**Tables 2**, **3**): the macroelements sodium (Na), potassium (K), calcium (Ca), magnesium (Mg), and phosphorus (P), and the microelements iron (Fe), copper (Cu), manganese (Mn), zinc (Zn), and boron (B). The values of total macroelements in florets and leaves were 46.107 g kg^-1^ DW and 73.47 g kg^-1^ DW, respectively, in control conditions (**Table 1**). Regarding the total microelements, they were 114.25 mg kg^-1^ DW in florets and 158.55 mg kg^-1^ DW in leaves in plants under control conditions (**Table 2**). In florets, K represented more than 78% of total macronutrients and Fe accounted for nearly 38% of the micronutrients. In leaves, K made up nearly 65% of the macronutrients and Fe more than 30% of the micronutrients. These results reveal that the purple cauliflower by-products were richer in nutrients than the floret. Although these values are within the expected range (Pennsylvania State University, College of Agricultural Sciences, 2024), the nutrient content found in the floret and leaves in this study is lower than that found in a previous study for white cauliflower floret and leaves (Collado-González et al., 2021a and (Collado-González et al., 2021b), but higher than that obtained in Australian white cauliflower florets and in florets from nine varieties of colored cauliflower (white, green, and purple) (Wang et al., 2022). These outcomes confirm that whole purple cauliflower is a rich source of minerals compared with other brassica species (Wang et al., 2022), and can be considered as intermediate regarding the mineral content of a wide variety of vegetables, fruits, and grains (Marles, 2017).

**Table 2:** Effect of the combination of inoculation with plant growth promoting bacteria and three different N concentrations in the nutrient solution on purple cauliflower macroelements (g kg^-1^ DW).

Treatment		Na	K	Ca	Mg	P
Floret						
100% N	No PGPB	1.31 ± 0.23^a^	36.04 ± 3.08^a^	1.46 ± 0.15^a^	1.40 ± 0.15^a^	5.90 ± 0.43^a^
	PGPB	1.50 ± 0.02^A^	38.86 ± 2.34^A^	1.63 ± 0.32^A^	1.57 ± 0.07^A^	6.56 ± 0.28^A^
60% N	No PGPB	0.99 ± 0.09^b^	34.73 ± 1.77^a^	1.31 ± 0.18^a^	1.39 ± 0.08^a^	5.72 ± 0.29^a^
	PGPB	1.23 ± 0.23^B^	36.91 ± 2.74^AB^	1.37 ± 0.32^A^	1.55 ± 0.16^A^	5.97 ± 0. 56^A^
30% N	No PGPB	0.94 ± 0.09^b^	34.23 ± 2.36^a^	1.51 ± 0.31^a^	1.44 ± 0.10^a^	5.68 ± 0.43^a^
	PGPB	1.21 ± 0.41^B^	34.20 ± 3.00^B^	1.74 ± 0.29^A^	1.36 ± 0.13^B^	5.26 ± 0.52^B^
Leaves						
100% N	No PGPB	2.30 ± 0.33^a^	47.61 ± 5.07^a^	13.05 ± 3.16^a^	3.23 ± 0.56^a^	7.28 ± 0.59^a^
	PGPB	3.26 ± 0.65^A^	61.35 ± 6.50^A*^	16.30 ± 3.58^A^	3.86 ± 0.56^A^	7.35 ± 0.45^A^
60% N	No PGPB	1.57 ± 0.34^b^	33.36 ± 4.64^b^	10.73 ± 2.58^ab^	2.64 ± 0.47^ab^	6.44 ± 0.66^ab^
	PGPB	1.80 ± 0.32^B^	42.07 ± 3.41^B*^	9.48 ± 1.10^B^	2.80 ± 0.30^B^	6.72 ± 0.49^A^
30% N	No PGPB	1.24 ± 0.21^b^	32.30 ± 3.43^b^	9.243 ± 2.24^b^	2.48 ± 0.57^b^	6.21 ± 0.79^b^
	PGPB	0.13 ± 0.27^B*^	37.57 ± 4.86^B^	8.48 ± 2.02^B^	2.36 ± 0.54^B^	5.67 ± 0.92^B^
Significance						
Nitrogen (N)	Floret	**	*	ns	ns	***
	Leaves	***	***	***	***	***
PGPB	Floret	**	*	ns	*	ns
	Leaves	**	**	ns	ns	ns
N × PGPB	Floret	***	ns	ns	**	*
	Leaves	**	**	ns	ns	ns

The data are presented as the treatment means (n = 5). Different lower case letters indicate significant differences between purple cauliflower plants under different dose of N supplied at p = 0.05 (Tukey’s test). Different upper case letters indicate significant differences between purple cauliflowers grown with or without PGPB inoculation. Asterisks indicate significant differences between inoculated and non-inoculated plants fertilized with the same N dose. Analysis of variance: ns, not significant; *p ≤ 0.05; **p ≤ 0.005; ***p ≤ 0.001. No PGPB, plants not inoculated with PGPB.

**Table 3:** Effect of the combination of inoculation with plant growth promoting bacteria and three different N concentrations in the nutrient solution on purple cauliflower microelements (mg kg^-1^ DW).

Treatment		Fe	Cu	Mn	Zn	B
Floret						
100% N	No PGPB	43.22 ± 7.47^a^	9.61 ± 1.52^a^	17.18 ± 2.09^a^	30.65 ± 6.62^a^	13.59 ± 0.87^a^
	PGPB	51.27 ± 9.85^A^	9.48 ± 2.02^A^	17.68 ± 1.51^A^	28.60 ± 2.40^A^	14.74 ± 1.16^A^
60% N	No PGPB	37.33 ± 3.13^ab^	7.84 ± 0.98^b^	18.31 ± 1.29^a^	25.78 ± 2.19^ab^	13.18 ± 1.21^a^
	PGPB	43.24 ± 3.86^B^	9.69 ± 1.32^A^	19.16 ± 1.89^A^	26.02 ± 3.35^A^	13.64 ± 1.18^AB^
30% N	No PGPB	36.27 ± 2.80^b^	7.94 ± 1.10^b^	18.59 ± 2.89^a^	23.71 ± 1.31^b^	14.49 ± 1.19^a^
	PGPB	37.40 ± 8.21^B^	9.05 ± 0.85^A^	18.51 ± 3.06^A^	20.99 ± 2.57^B^	12.88 ± 1.01^B^
Leaves						
100% N	No PGPB	48.08 ± 8.55^a^	8.96 ± 1.06^a^	51.66 ± 9.37^a^	20.28 ± 4.31^a^	29.57 ± 4.35^a^
	PGPB	62.64 ± 11.19^A*^	9.95 ± 1.68^A^	54.51 ± 11.75^A^	19.93 ± 2.92^A^	34.21 ± 3.33^A^
60% N	No PGPB	39.32 ± 3.66^b^	8.80 ± 1.40^a^	51.07 ± 11.26^a^	18.84 ± 3.37^a^	25.98 ± 2.71^a^
	PGPB	44.05 ± 3.23^B^	8.56 ± 1.02^AB^	43.21 ± 8.77^A^	19.01 ± 3.30^A^	26.83 ± 2.93^B^
30% N	No PGPB	32.25 ± 4.18^b^	8.98 ± 1.46^a^	40.92 ± 7.87^a^	16.40 ± 3.65^a^	26.45 ± 2.93^a^
	PGPB	43.15 ± 5.25^B*^	7.67 ± 1.11^B^	42.37 ± 9.01^A^	17.54 ± 4.01^A^	22.91 ± 3.13^C^
Significance						
Nitrogen (N)	Floret	***	ns	ns	***	ns
	Leaves	***	ns	*	ns	***
PGPB	Floret	ns	*	ns	ns	ns
	Leaves	ns	ns	ns	ns	ns
N × PGPB	Floret	ns	ns	*	ns	*
	Leaves	***	ns	ns	ns	*

The data are presented as the treatment means (n = 5). Different lower case letters indicate significant differences between purple cauliflower plants under different dose of N supplied at p = 0.05 (Tukey’s test). Different upper case letters indicate significant differences between purple cauliflowers grown with or without PGPB inoculation. Asterisks indicate significant differences between inoculated and non-inoculated plants fertilized with the same N dose. Analysis of variance: ns, not significant; *p ≤ 0.05; ***p ≤ 0.001. B, boron; Cu, cupper; Fe, iron; Mn, manganese; No PGPB, plants not inoculated with PGPB; Zn, zinc.

As a result of the reduced N supply, variations in the concentrations of most of the cations studied were found, depending on the part of the purple cauliflower plant studied. In this sense, Na and Fe were the cations that showed a significant decrease in the floret and leaves as the N supply decreased. Conversely, B and Mn were not affected by the reduction in N fertilization (**Table 3**). The contents of K, Ca, Mg, and P decreased in the leaves, but did not change in the floret, as the N supply decreased. In contrast, the contents of Cu and Zn in the leaves were not affected by N restriction, but decreased in the floret (**Tables 2**, **3**). These results are consistent with those previously reported in other studies with lettuce and white cauliflower, where the vegetables were subjected to stress due to reduced mineral fertilizer applications and high temperatures, respectively (Collado-González et al., 2021b; Consentino et al., 2022; Ikiz et al., 2024). This effect of mineral content can be attributed to the fact that some macro and microelements are involved in the antioxidant system of the plants (Collado-González et al., 2024). Hamner et al. (2017) established a relationship between N uptake and the increased uptake of the plant’s other nutrients.

*Azotobacter salinestris* CECT 9690 elicited the accumulation of K (29%) and Fe (30%) in leaves. Plants inoculated with this PGPB and fertigated with 30% of N led to a reduction of 90% for Na in leaves and an accumulation about 29 of percentage for Na in floret. Due to this interaction, it was also observed an increase of 26% for K and with 30% of N supplied the Fe increased about 34% in leaves (**Tables 2**, **3**). ANOVA did not reveal a significant effect of the interaction N × PGPB on the other cations studied. Initially, it was expected that all cations would respond homogeneously to the action of the PGPB. Nevertheless, the response obtained was varied. According to Ikiz et al. (2024), this type of response is very likely due to the interaction between the specific cauliflower cultivar and PGPB. The higher accumulation of K and Fe in the cauliflower by-products shows that the PGPB promoted their accumulation in plant tissues. Radhakrishnan and Lee (2016), in a study on lettuce, associated the greater accumulation of these nutrients due to the action of *B. methylotrophicus* KE2 with a catalyzed metabolism of proteins, enzymes, lipids, and nucleic acids. On the other hand, Abdel Latef et al. (2020) reported in a study with maize a direct correlation between the ion Na and MDA, indicating that a reduction of Na involves a reduction of oxidative stress. In the current work, a reduction of Na was observed in leaves of plants fertigated with a supply of 30% of N and inoculated with PGPB.

### 3.4 Sugars content

**Figure 2:**
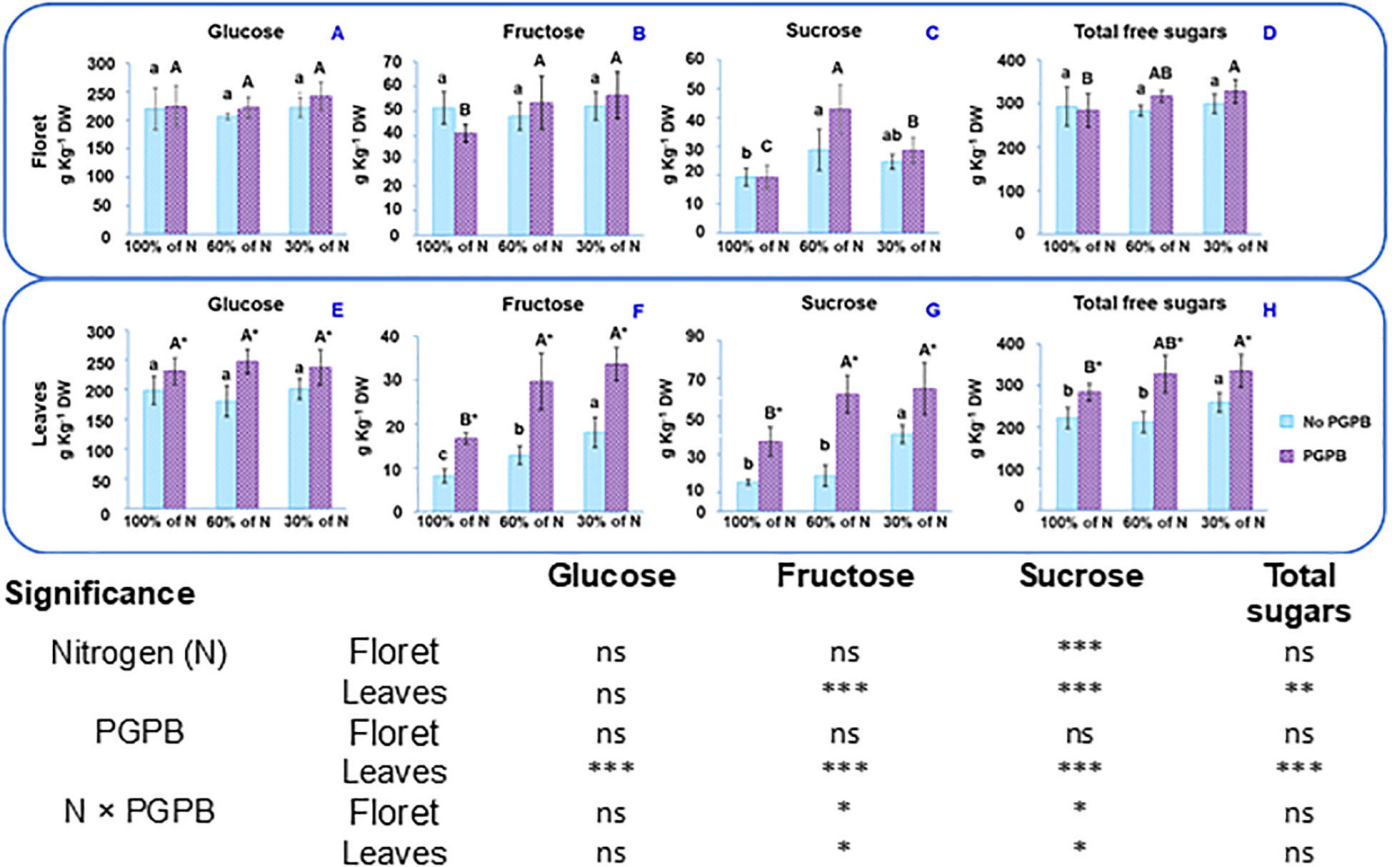
Effect of the combination of inoculation with A. salinestris and three different N concentrations on purple cauliflower sugars of the floret **(A-D)** and leaves **(E-H)**. The data are presented as the treatment means (n = 5). Different lower case letters indicate significant differences between purple cauliflower plants fertilized with different N% supplies, at p = 0.05 (Tukey’s test). Different upper case letters indicate significant differences between purple cauliflower plants fertilized with different N% supplies and inoculated with PGPB. Analysis of variance: ns, not significant; *p ≤ 0.05; **p ≤ 0.005; ***p ≤ 0.001. No PGPB, plants not inoculated with PGPB.

In this study, the presence of the three main sugars found in purple cauliflower was determined: glucose, sucrose, and fructose (**Figures 2A–H**). Glucose was the most abundant in all parts of the plants and sucrose the least. Similar results were reported for white cauliflower (Bhandari and Kwak, 2015; Collado-González et al., 2021b).

Comparing the total free sugars contents of the floret and leaves, it can be observed in **Figure 2** that although the floret has a higher sugar content, the leaves also show a high sugar content, close to the values obtained for the floret. These results are supported by the findings of various authors in cauliflower (Bhandari and Kwak, 2015; Collado-González et al., 2021a and Collado-González et al., 2021b; Ingallina et al., 2023). For the distinct N doses in the nutrient solution, the total free sugars content in the floret oscillated between 292.7 g kg^-1^ DW and 299.1 g kg^-1^ DW and in leaves from 221.9 g kg^-1^ DW to 259.6 g kg^-1^ DW. As can be seen in **Figure 2**, total free sugars in florets were not changed as a result of the N deficiency, but they were increased in leaves as N deficiency intensified. The study shows that the concentration of each sugar in the floret and leaves from plants at different N doses behaved differently. In this sense, in the floret the glucose and fructose contents were not significantly affected by the N deficiency (**Figure 2**). However, **Figure 2** shows that plants fertilized with 60% N had florets with the highest sucrose concentration, whilst the florets from control plants presented the lowest sucrose content. In leaves, glucose did not exhibit significant differences due to the N supply and fructose and sucrose showed a dose-dependent behavior, the highest fructose and sucrose contents occurring with the 30% N supply (**Figure 2**). Thus, the N dose influenced the contents of both total and individual sugars. Some authors have suggested that both too low and too high a supply of nitrate to plants significantly affect them (Albornoz, 2016; Zhang et al., 2020, 2021). An optimal N supply promoted the synthesis of reducing sugars and the metabolic utilization capacity in wild apple tree shoots and also accelerated the carbon flow to meet the sugar demands necessary for shoot growth (Zhang et al., 2021).

When the activity of *A. salinestris* is also considered, the total sugar concentration ranged from 284.2 g kg^-1^ DW to 327.6 g kg^-1^ DW in florets and from 284.5 g kg^-1^ DW to 335.4 g kg^-1^ DW in leaves. As shown in **Figure 2**, the combination of both factors (limited N and PGPB inoculation) resulted in a significant increase in the fructose and sucrose contents, in both parts of the plants. These results were expected, since previous studies have found that PGPB bacteria, such as *A. brasilense* Ab-V5 and *Herbaspirillum* sp. AP21, among others, showed greater efficiency in plants under reduced N supply (Calzavara et al., 2017; Aquino et al., 2021; Pardo-Díaz et al., 2021). However, our outcomes showed that when only the effect of PGPB inoculation is considered, only the individual and total free sugars contents in leaves showed significant differences. This can be attributed to the increase in K concentration and the reduction in the N:K ratio in the leaves with respect to the floret. Not only is K a fundamental nutrient for plant growth, it also plays an essential role in many metabolic processes, such as protein synthesis, the translocation of carbohydrates and assimilates within the plant, and the accumulation of high-molecular-weight carbohydrates, which are important for the formation and development of plant fruits (Metwaly, 2017). Tallarita et al. (2024) claimed that an adequate N:K ratio in cauliflower is crucial for achieving good vegetative growth and ensuring strong stress resistance. In this sense, these authors suggested that an N:K ratio between 0.8 and 1.2 promoted greater plant development and improved quality in cauliflower. This N: K ratio depends on several factors, including the plant genotype, environmental conditions and the agronomic practices to which the crop is subjected (Ali et al., 2019). As previously described, in the current study, the inoculation of purple cauliflower plants with *A. salinestris* resulted in a higher concentration of K in the leaves than in the florets. Additionally, an N:K ratio of around 0.56 was obtained in the leaves, while a considerably higher ratio was found in the purple cauliflower floret, ranging from 0.70 (30% of N supplied) to 0.95 (100% of N supplied). For this reason, the floret showed greater resilience to N deficiency stress, while the leaves were more susceptible to the action of the bacteria.

### 3.5 Protein content

**Figure 3:**
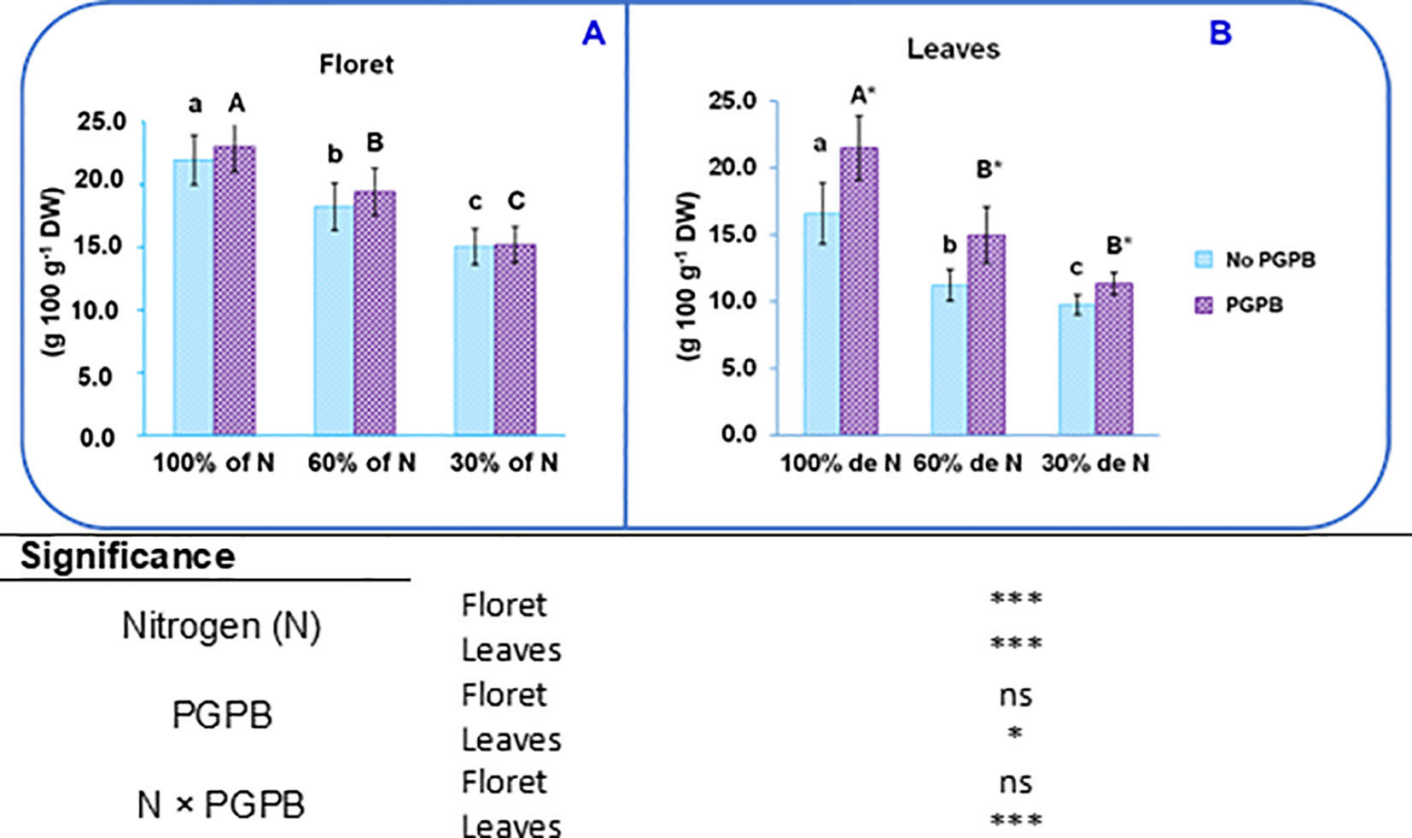
Effect of the combination of inoculation with **(A)** salinestris and three different N concentrations on proteins in the purple cauliflower floret **(A)** and leaves **(B)**. The data are presented as the treatment means (n = 5). Different lower case letters indicate significant differences between purple cauliflower plants fertilized with different N% supplies, at p = 0.05 (Tukey’s test). Different upper case letters indicate significant differences between purple cauliflower plants fertilized with different N% supplies and inoculated with PGPB. Analysis of variance: ns, not significant; *p ≤ 0.05; ***p ≤ 0.001. Not PGPB, plants not inoculated with PGPB.

The protein content in purple cauliflower tissues under control conditions varied from 16.6 g 100 g^-1^ DW in the leaves to 22.0 g 100 g^-1^ DW in the floret (**Figure 3**). The value obtained for the floret of this study was close to that found by Kapustcha et al. (2019), who indicated that the raw floret of purple cauliflower contained 25.7 g of protein per 100 g of DW. The value obtained for leaves was lower than that indicated by Collado-González et al. (2021a) in a study carried out with white cauliflower.

The dose of N provided in the nutrient solution affected the protein content in the different purple cauliflower tissues. The protein content in the floret showed a significant decrease of 17% when 60% N was supplied and of 32% at 30% N supply, with respect to the control. Likewise, important reductions of 32% and 41% were observed in leaves when 60% and 30% N were supplied, respectively (**Figure 3**). The degradation of proteins is one of the rapid responses of plants when they are exposed to different abiotic stresses; for instance, N deficiency. Nitrogen deficiency induces an increased production of reactive oxygen species (ROS), which cause cellular damage that manifests as the degradation of some biomolecules such as proteins (Tewari et al., 2021; Collado-González et al., 2024).

Additionally, as was the case with sugars, the protein content in the leaves of purple cauliflower plants was increased by the action of *A. salinestris*: by about 33% when 60% N was supplied and by 16% at 30% N supply (**Figure 3**). Some authors have reported that different kinds of PGPB promoted the soluble protein content in several plant species, such as maize, sorghum, and celery (Abdel Latef et al., 2020; Aquino et al., 2021; Collado-González et al., 2024). The fact that here the protein content in leaves was affected by the PGPB highlights the greater susceptibility of the leaves to the negative effects of nutritional stress, which are mitigated by the beneficial action of the PGPB. It is worth mentioning that leaf tissues show greater susceptibility to ionic stress than most other plant tissues (Gupta et al., 2022). In this sense, it was expected that the N: K ratio were lower in leaves (0.56) than in florets (ranged from 0.70 to 0.95). As mentioned above, this N: K ratio is influenced by climatic conditions and the agronomic practices applied to the crop (Ali et al., 2019). The beneficial effect of PGPB on protein content is likely to be a direct result of their ability to fix N, but it can also be an indirect consequence of their capacity to synthesize osmoprotectants (proline, choline and trehalose), volatile compounds (i.e., 2R,3Rbutanediol), phytohormones (IAA, gibberellins and cytokinins) or produce 1-aminocyclopropane-1-carboxylic acid (ACC) deaminase activity (Abdel Latef et al., 2020; Lephatsi et al., 2021; Consentino et al., 2022).

## 4 Conclusions

The use of A. *salinestris* bacteria improved the plant growth parameters and dry matter content of purple cauliflower under N deficiency. The inoculation of plants with *A. salinestris* also boosted their nutritional status, particularly for the by-products of purple cauliflower in terms of sugars and proteins. This highlights the greater resilience of the florets to nutritional stress and the higher susceptibility of the leaves to the detrimental effects of N deficiency. This could be due to the fact that the florets showed N:K ratio values ranging from 0.7 (30% of N supplied) to 0.95 (100% of N supplied), while the leaves displayed lower ratios (0.47 and 0.56 for N inputs of 30% and 100%, respectively). Neither AA nor total phenolic content was affected by the combined action of the low N dose and the application of these PGPB. As expected, purple cauliflower had a higher AA than white cauliflower.

Worthy of note is the increase in the K and Fe concentrations in the by-products due to the action of *A. salinestris*. These results highlight the improvement in both growth and nutritional quality, particularly in the by-products of purple cauliflower under optimal and low N supply, due to the action of these PGPB. Moreover, this work also underlines the potential to boost the valorization of by-products from this type of cauliflower. Thus, the application of this technology and crop management can be beneficial for growers, the cosmetics and nutraceutical industries, and environmental sustainability.

## Data Availability

The original contributions presented in the study are included in the article/supplementary material. Further inquiries can be directed to the corresponding authors.
